# Osmotic Engine: Translating Osmotic Pressure into Macroscopic Mechanical Force via Poly(Acrylic Acid) Based Hydrogels

**DOI:** 10.1002/advs.201700112

**Published:** 2017-05-30

**Authors:** Lukas Arens, Felix Weißenfeld, Christopher O. Klein, Karin Schlag, Manfred Wilhelm

**Affiliations:** ^1^ Institute for Technical Chemistry and Polymer Chemistry (ITCP) Karlsruhe Institute of Technology (KIT) 76131 Karlsruhe Germany

**Keywords:** energy recovery, hydrogels, osmotic engine, poly(acrylic acid), polyelectrolytes

## Abstract

Poly(acrylic acid)‐based hydrogels can swell up to 100–1000 times their own weight in desalinated water due to osmotic forces. As the swelling is about a factor of 2–12 lower in seawater‐like saline solutions (4.3 wt% NaCl) than in deionized water, cyclic swelling, and shrinking can potentially be used to move a piston in an osmotic motor. Consequently, chemical energy is translated into mechanical energy. This conversion is driven by differences in chemical potential and by changes in entropy. This is special, as most thermodynamic engines rely instead on the conversion of heat into mechanical energy. To optimize the efficiency of this process, the degree of neutralization, the degree of crosslinking, and the particle size of the hydrogels are varied. Additionally, different osmotic engine prototypes are constructed. The maximum mean power of 0.23 W kg^−1^ dry hydrogel is found by using an external load of 6 kPa, a polymer with 1.7 mol% crosslinking, a degree of neutralization of 10 mol%, and a particle size of 370–670 µm. As this is achieved only in the first round of optimization, higher values of the maximum power average over one cycle seem realistic.

## 1 Introduction

A charged polymeric network forms a hydrogel in water due to the difference in the osmotic pressure between the network and the surrounding solvent.^1^ Poly(acrylic acid)‐based hydrogels can swell up to 100–1000 times their own weight in desalinated water, while the swelling in saline solutions, e.g., 4.3 wt% NaCl having a similar chemical potential as seawater, is typically a factor of 2–12 lower.^2^ Such hydrogels are called superabsorbent materials and are employed in large quantities in sanitary products like diapers.^3^

If a hydrogel is swollen in a saline solution, only part of the salt ions will enter into the polymeric network because of Coulombic repulsion with the negatively charged polymer chains.^4^ After removing the hydrogel from the saline solution, a force can be applied to squeeze the water out of the hydrogel. As a result, water with a lower salt content is obtained. This new process opened up the possibility to desalinate saltwater via polymeric hydrogels.^5^, ^6^

The idea of this publication is to invert this desalination process. Consequently the hydrogel is exposed to fresh and seawater in an alternating fashion and the swelling–deswelling process is used to generate mechanical forces. In this manner, the cyclical swelling in deionized water and shrinking in saline solution causes a mechanical movement. This motion is used to move a weight upward and downward in a similar way to how a piston moves in a combustion motor. Thereby chemical energy is translated into mechanical energy.

The concept of using differences in chemical potential and changes in entropy, to translate chemical energy into mechanical energy is not new. In 1962, Kuhn first published the idea of a pH‐muscle. The pH‐muscle works by first swelling a polyelectrolyte filament in a cylinder filled with water.^7^ Next, a weight was fixed to the filament and it was then transferred into a cylinder full of NaOH, which stretched the filament. Afterward, by putting the filament into a cylinder filled with HCl the filament shrank and pulled the weight upward.

In 1966, Katchalsky and co‐workers introduced a concept to use solutions with different salt concentrations, to obtain mechanical energy.^8^ They developed a mechanochemical engine in which a crosslinked collagen string is coiled around several drive pulleys. One part of the string is placed in a concentrated LiBr solution, whereas the other end is in a diluted LiBr solution. The string contracts in the concentrated solution, but expands in the dilute solution. Due to these processes, a torque is generated that moved a weight upward.

One of the most advanced methods that use differences in chemical potentials, to convert chemical energy into mechanical energy on a large scale is based on the idea of Loeb to use membranes and the principal of osmosis.^9^ The Norwegian company Statkraft used this concept in 2009 to build the first prototype of an osmotic power plant with an energy output between 2 and 4 kW in Tofte, Norway.^10^ To achieve this, two chambers were build that are separated by a semipermeable membrane. One chamber contains seawater and the other chamber contains river water, which has a lower salinity. Due to the osmotic pressure difference, river water flows into the chamber containing seawater causing the pressure to increase and drive a turbine to produce finally electrical energy.^11^ However, the project was cancelled at the end of 2013 because the osmotic pressure power plant was economically not competitive with other power plants and it was not expected that this would change in the near future.^12^

In 2014, Logan and co‐workers demonstrated the proof of principal that shrinking and swelling of a polyelectrolyte hydrogel can also be used to get mechanical energy by chemical potential differences.^13^ They generated 0.1 W kg^−1^ (dry) of a poly(acrylic acid) hydrogel with a degree of crosslinking (DC) of 2.0 mol% and a degree of neutralization (DN) of 60 mol%.

Logan and co‐workers focused on process parameters to study the energy production in an osmotic engine and did not investigate the effect of different molecular structures. Therefore, it is not known how chemical parameters such as the DC, the DN, and the particle size can influence the obtained power. A theoretical prediction, of which chemical structure of the hydrogel will perform best, is not straightforward, since the energy production of the osmotic engine depends on a complex interaction between mechanical strength, maximum degree of swelling in deionized and salt water, as well as the corresponding swelling kinetics of the hydrogels. Hence, the goal of the work presented here is to investigate and screen the effect of synthetic parameters.

First, we varied the chemical composition of the hydrogel by changing the DC and DN to find the settings that optimize power generation. Second, we evaluated different engine prototypes and, third, we showed that repeated swelling and deswelling cycles are possible without damaging the hydrogel.

### 1.1 Swelling

Linear and uncharged polymer chains dissolve in an adequate solvent due to favorable enthalpic but mainly entropic effects caused by mixing.^14^ However, a polymer network is composed of covalent crosslinked polymer chains so that the network can only swell in a good solvent instead of dissolving.^15^ In case of a charged polymer network, the charges along the polymer chain repel each other (Coulomb repulsion) and the chains stretch further when swelled. Consequently, the maximum degree of swelling is larger for polymers with high charge density relative to uncharged polymers. The volume increase caused by swelling can be expressed as the osmotic pressure Π of an ion carrying solution as given by Equation (1), 2

(1) $$\Pi =\frac{n}{V_{m}}\times iRT$$

In this equation, *n* are the moles of dissolved molecules (e.g., NaCl), *V*_m_ is the volume of solvent, *R* is the universal gas constant, *T* is the absolute temperature, and *i* is the van't Hoff factor (e.g., *i* = 2 for NaCl). As a consequence of Equation (1), the osmotic pressure Π increases linearly with increasing salt concentration at low concentrations.

### 1.2 Optimization Criteria

To rate the performance of an osmotic engine, the obtained work, respective power was measured and calculated. The work performed by any motor can be expressed as

(2) W=∫F×dx

or, as the stress σ is the force normalized by the area

(3) $$\sigma =\frac{F}{A}$$

which can also be expressed for an elastic material with a Young's modulus *E* as

(4) σ=∫E×ε×dε

to result in

(5) W=∫A×E×ε×dε

where *A* is the area, *W* is the work, *F* is the force, *E* is the Young's modulus of elasticity, ε is the normalized elongation, and d*x* is the change in height due to swelling of the hydrogel.

Power can be defined as the current power *P*(*t*) or the mean power $\bar{P}$ per cycle. The current power is the power generated at a specific point in time. Mean power refers to the average power over one cycle, which is here defined as the time starting from when the swollen polymeric hydrogel reaches equilibrium in a 4.3 wt% NaCl solution to the time when the hydrogel swollen in deionized water nearly reaches equilibrium. As there were some experiments where only the initial and final values were measured, the average of these two values was used for determining the mean power $\bar{P}$ in Equation (6)

(6) $$\bar{P}=\frac{\Delta W}{\Delta t}=m\times g\times \frac{\Delta h}{\Delta t}$$

where Δ*W* is the work, Δ*t* is the time required for one cycle, *m* is the mass moved by the piston, *g* is the gravitational constant of 9.81 $\frac{m}{s^{2}}$ and Δ*h* is the lifting height.

## 2 Results and Discussion

In this publication, poly(acrylic acid)‐based hydrogels with different DN and different DC were evaluated with respect to their performance in an osmotic motor. The influence of the external load, respective pressure, was investigated as well as the effect of particle size on the resulting mean power production. The repeatability of the swelling and shrinking cycles of the polymeric hydrogel and the effect of drainage materials were also evaluated.

### 2.1 Particle Size

**Figure 1:**
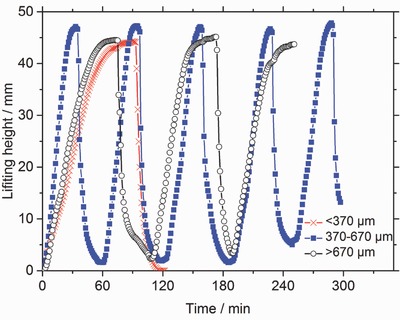
Several swelling and shrinking cycles for the commercial sample RD 474 with different particle sizes as measured in Osmotic Engine 1. Hydrogels with a particle diameter of 370–670 µm show the shortest cycling time of about 60 min, while the cycling times for particles smaller than 370 µm and larger than 670 µm increase to 120 and 110 min, respectively.

The swelling of hydrogels can be divided into two separate steps. First, water flows into the spaces between the particles. Second, water diffuses inside the particles. If the particles are too small, water diffusion into the outer particles is so fast that the flow of water to the inner particles is blocked. This is called gel‐blocking and is a major problem in hygiene products like diapers.^16^ Different particle sizes (dry), >670, 370–670, and <370 µm in diameter, were evaluated with respect to their swelling kinetics, as shown in **Figure** **1**. For particles between 370 and 670 µm in diameter, the time to complete one power cycle was about 60 min. For particles smaller than 370 µm and larger than 670 µm, this time increased to 120 and 110 min, respectively. The slower swelling times for hydrogels with particles smaller than 370 µm can be explained by gel‐blocking. However, for hydrogels with particles larger than 670 µm, it is possible that the longer swelling times was caused by the large interstitial space between particles causing the particles to expand (deform) radially to fill the space before then expanding primarily axially to move the weight. These results agree with previous work by Wack and Ulbricht who also found experimentally that the best particle size with respect to swelling kinetics was between 300 and 600 µm.^17^ Therefore, the particle size was kept within this range, 370–670 µm, for all further experiments.

### 2.2 Degree of Neutralization

**Figure 2:**
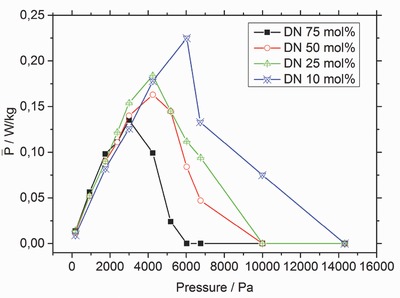
Influence of the degree of neutralization (DN) on the obtained mean power over one cycle in Osmotic Engine 2. The degree of crosslinking was kept constant at 1.7 mol%, while the external load was varied between 0 and 16 000 Pa for the four samples. A lower value of DN resulted in a higher mean power at higher external loads. The lines are only drawn as a guide for the eyes.

Charges inside a hydrogel are responsible for its swelling behavior. The more charges the polymer contains, the higher is its polarity. Therefore, the degree of swelling increases monotonic, but not linear with increasing DN if no external pressure is applied. Additionally, the ratio between the degree of swelling in deionized water over salt water decreased with increasing DN, as seen in **Table** **1**. One of the goals of this work was to evaluate how the DN (see Equation (7)) affects the resulting power production. Therefore, samples with different DN values (10, 25, 50, and 75 mol%), but the same DC of 1.7 mol%, were tested and their power production per cycle was measured in Osmotic Engine 2. **Figure** **2** displays the power output as a function of DN and the applied pressure (external load X). If the external load is small, the power is independent of the DN, while lower values of DN, fewer charges along the polymer chain, is advantageous at higher external loads. One possible explanation is that a more charged hydrogel has a higher degree of swelling, which causes the hydrogel to become mechanically weaker and less able to move the heavier weights.^16^ Therefore, a lower DN results in a better performance at higher external loads.

**Table 1:** Overview of the self‐synthesized hydrogels with varying degrees of crosslinking (DC) and degrees of neutralization (DN). The particle size was kept within the range of 370–670 µm and the degree of swelling *Q* was determined in both deionized water (DI) and in a 4.3 wt% NaCl solution as a model for seawater

Sample	DC [mol%]	DN [mol%]	*Q* [g g^−1^] (DI)	*Q* [g g^−1^] (4.3 wt% NaCl)
PAA_DC_0.3_DN_75	0.3	75	271.5	21.6
PAA_DC_1.0_DN_75	1.0	75	101.2	15.5
PAA_DC_1.4_DN_75	1.4	75	66.5	12.8
PAA_DC_1.5_DN_75	1.5	75	57.9	12.4
PAA_DC_1.6_DN_75	1.6	75	56.3	12.2
PAA_DC_1.7_DN_75	1.7	75	51.4	11.9
PAA_DC_1.7_DN_50	1.7	50	48.4	9.6
PAA_DC_1.7_DN_25	1.7	25	44.2	5.1
PAA_DC_1.7_DN_10	1.7	10	30.5	2.5
PAA_DC_2.0_DN_2.5	2.0	2.5	10.6	4.2
PAA_DC_2.0_DN_5.0	2.0	5.0	15.6	4.8
PAA_DC_2.0_DN_10	2.0	10	27.4	5.1
PAA_DC_2.5_DN_75	2.5	75	32.5	10.3
PAA_DC_2.5_DN_10	2.5	10	20.2	2.3
PAA_DC_3.4_DN_75	3.4	75	23.5	8.1
RD 474	N/A	N/A	72.0	15.2

### 2.3 Degree of Crosslinking

The DC determines the average mesh length of the network, hence it influences both the absorbency and the mechanical strength. A denser network leads to a lower water uptake *Q* (see Table 1) and mechanically more stable hydrogels.^18^, ^19^, ^20^, ^21^ Hydrogels with a DC between 0.3 and 3.4 mol% and a DN of 75 mol% were evaluated with respect to their maximum mean power $\bar{P}$. Additionally, the applied pressure was varied over the range of 130–26 000 Pa because the piston weight affects the cycle time.

**Figure 3:**
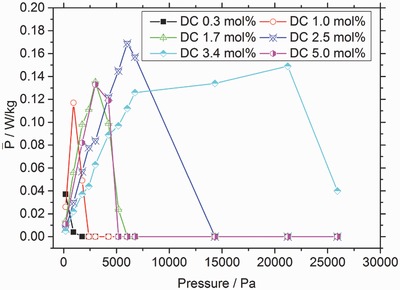
Influence of the degree of crosslinking (DC) on the obtained power in Osmotic Engine 2 as a function of the external load at a constant DN of 75 mol%. The maximum power of each sample was reached at higher external loads if a higher DC was used. The uncertainty for repeating a measurement three times was typically below 8%. The lines are only drawn as a guide for the eyes.

For each DC there is a specific pressure at which the maximum power is reached, as seen in **Figure** **3**. This specific pressure increases with increasing DC because these hydrogels have a higher *E*‐modulus and are able to move heavier weights, but mainly over shorter distances.

### 2.4 Influence of the Osmotic Engine Prototype on Power Production

**Figure 4:**
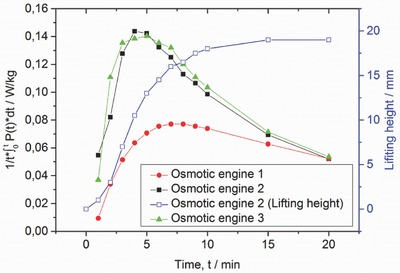
The accumulative of the current power is compared for the different osmotic engine prototypes with the commercial sample RD 474. The energy production drawn on the *y*‐axis is the time average energy production until the point *t*. This energy production depends on the lifting speed (dilation per time). The dilation is shown additionally for Osmotic Engine 2 in open squares for a better understanding of the power evolution. While Osmotic Engine 1 and 2 perform better at the beginning, the power production becomes similar after 20 min for all three prototypes.

In addition to the investigation of chemical parameters toward the obtained mean power $\bar{P}$, three different prototypes of a potential osmotic engine were built and compared using sample RD 474 (see **Figures** **4** and 7). During these experiments, the amount of the dry polymer was normalized to the piston area to get the same thickness of the gel layer in all three engines. This led to a required polymer mass of 6.5 g in Prototype 1, 0.56 g in Prototype 2, and 1.0 g in Prototype 3. Additionally, the external load was kept constant at 1800 Pa (corresponding to a 1.452 kg load in Prototype 1, a 0.125 kg load in Prototype 2, and a 0.226 kg load in Prototype 3). The results in Figure 4 show that especially the Prototype 1 has a lower initial power output *P*(*t*), most probably caused by friction of the quadratic piston. This effect is temporary as the obtained, accumulated power is similar after 20 min for all three prototypes.

### 2.5 Reusability

**Figure 5:**
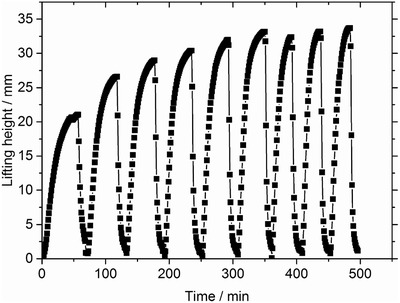
The sample PAA_DC_2.0_DN_5.0 experienced nine swelling and shrinking cycles in Osmotic Engine Prototype 1. The swelling and shrinking cycles could be repeated and later cycles led to a 40% higher lifting height and a 60% reduction in the cycle time. This is probably due to sol extraction of polymer chains that were not crosslinked.

If polyelectrolyte hydrogels are used as a material in an osmotic engine, the structure of the hydrogel must sustain several swelling and shrinking cycles without damage. The sample PAA_DC_2.0_DN_5.0 was used to evaluate the reusability of the swelling and shrinking behavior over nine cycles. A lifting height of 21–29 mm was obtained within the first three cycles, whereas a lifting height of 30–34 mm was obtained between cycles four and nine, as seen in **Figure** **5**. The process of swelling and shrinking seems not to damage the hydrogel and the process levels off to a steady lifting height. The increase in the lifting height for the first three cycles (Figure 5) may be caused by the extraction of the “sol” polymer chains, which are not bonded to the crosslinked hydrogel. Extraction of the sol from the polymer network leads to both a higher lifting height and a faster swelling, consequently, a larger power production. After the first three cycles, the lifting height increased by 40%, while the time per cycle was reduced by 60%. These changes increased the average power production by a factor of 3.5 in total for this specific sample and shows that the energy productivity shown in the previous sections should be rather seen as lower estimation.

The argument that the increasing energy productivity is related to extraction of sol is also supported by Figure 1 where several cycles for the commercial sample RD 474 are shown. The commercial sample has a very small amount of extractables below 2 wt%. Consequently, the energy productivity is nearly unaffected within the first cycles as seen in Figure 1. Hence, the sol fraction is crucial for the increased energy productivity which depends on the synthesis conditions, in particular the degree of crosslinking. In previous work, the sol fraction has been determined for samples which were synthesized by the same synthesis procedure.^18^ In that work, no significant change of the sol content was found for samples with a DC ≥ 1 mol%, which had between 7 and 8 wt% extractables. Therefore, it is likely that the increasing energy production for the samples (with a DC ≥ 1 mol%) in the previous sections will be in the same order of magnitude as the sample PAA_DC_2.0_DN_5.0, while the improvement of the sample with a DC of 0.3 mol% (with 12 wt% sol) might be even higher.

### 2.6 Drainage

The swelling kinetics is one essential point for generating a larger amount of energy with the osmotic motor as the power is determined by the work per unit of time. Gel‐blocking decelerates the swelling of hydrogels. Accordingly, different drainage materials such as polyurethane foam, molecular sieves, metal sieves, silicone tubes, fiberglass, clay granulates, and diaper fleece were evaluated to determine which material led to the quickest penetration of the inner hydrogel particles to achieve the fastest swelling times.

The largest increase in the hydrogel swelling rate (commercial sample RD 474) was obtained with diaper fleece (polypropylene) as the drainage material. The pure hydrogel (reference) produced 0.04 W kg^−1^ mean power within 24 min, whereas the polymer mixed with diaper fleece resulted in a maximum mean power of 0.09 W kg^−1^ within 8 min as measured in Osmotic Engine Prototype 1. Faster swelling kinetics were also seen in Osmotic Engine 3. The maximum mean power was 0.06 W kg^−1^ for the reference over 10 min without drainage material and was 0.15 W kg^−1^ over 5 min with diaper fleece as the drainage material. This demonstrates that the fleece drainage systems were more efficient independent of the osmotic engine prototype. For Osmotic Engine Prototype 1 and 3, the mean power over one cycle increased by 125% and 150%, respectively.

As an estimate for the minimum time period Δ*t*, Ficks second law and a 3D diffusion with a diffusion coefficient of water (*D =* 2 × 10^−9^ m^2^ s^−1^) is assumed. This leads to about 350 µm mean square displacement if 〈*r^2^*〉 = 6 × *D* × *t* and 10 s diffusion time is assumed. This is a clear indication that optimized drainage could gain further 1–2 decades in power production.

## 3 Conclusions and Outlook

Polyelectrolyte hydrogels can translate chemical energy into mechanical energy driven by a chemical potential difference via the corresponding osmotic pressure difference in deionized water and salt water. The purpose of this publication is to demonstrate a proof of principle for this concept.

The use of poly(acrylic acid) hydrogels in an osmotic engine was evaluated to determine the maximum power production. The effect of synthetic parameters such as the DC, the DN and the particle size was investigated. A particle size between 370 and 670 µm led to the fastest swelling. A lower DN resulted in a higher mean power when larger external loads were used. The effect of the DC also depended on the magnitude of the external load. A higher DC performed better when larger weights needed to be lifted. The maximum mean power produced so far was 0.23 W kg^−1^ of dry polymer. This power was obtained using a sample that had a DC of 1.7 mol% and a DN of 10 mol% using 0.25 g dry polymer particles with a diameter between 370 and 670 µm and an external load of 6000 Pa.

The cyclic performance of the osmotic engine was also investigated as this result is important for its potential commercial use. The hydrogels showed no detectable damage after nine cycles of shrinking and swelling. One of the main challenges of this concept is slow speed of the hydrogel swelling and shrinking due to gel‐blocking. To minimize gel‐blocking, different drainage materials were employed. A polypropylene fleece used in diapers increased the maximum mean power by up to 150%. A further reduction in gel‐blocking may be possible if the voids between particles are maximized by using spherical particles with a small size distribution. These types of particles can be synthesized, e.g., by a droplet‐based microfluidic templating process.^22^, ^23^

An attempt to decrease the cycling time by optimizing the cycle to the times where swelling and shrinking were at their fastest, but not at equilibrium, did not show an improvement in the resulting power.

While these early results show promise, it should also be noted that the effect of multivalent cations in natural sea water must be considered as this reduces the degree of swelling due to additional ionic crosslinks.^24^, ^25^ However, with even more effective hydrogels, full chemical and engineering optimization, it is possible to imagine these types of osmotic engines in river deltas as a steady source of renewable energy since nearly unlimited salt and fresh water is available in this specific locations.

## 4 Experimental Section

**Figure 6:**
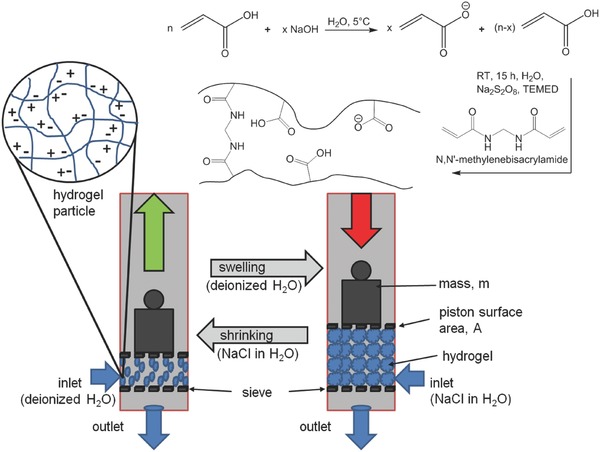
Scheme showing the conversion of chemical energy into mechanical energy via changes in salinity. Left) A hydrogel swollen in a 4.3 wt% NaCl solution, as a model for seawater, is exposed to fresh water. Driven by the osmotic pressure, fresh water enters the hydrogel and the resulting further swelling causes a weight to move upwards. Right) The swollen hydrogel is then again exposed to a 4.3 wt% NaCl solution causing the polymeric hydrogel to shrink. This process can be repeated.

*Synthesis*: The hydrogels were synthesized by free‐radical polymerization of acrylic acid with *N*,*N*′‐methylenebisacrylamide as the crosslinking agent and sodium persulfate (Na_2_S_2_O_8_) as the initiator in an aqueous solution at room temperature for 15 h (see **Figure** **6**).

First, acrylic acid (25 wt% aqueous solution) was neutralized to a final degree of 10–75 mol% with a 33 wt% NaOH solution while being cooled in an ice bath. Next, 1–3.4 mol% crosslinker and 0.2 mol% initiator (based on the moles of acrylic acid) were added. The resulting solution was then sparged with nitrogen gas for 45 min. Afterward, 2 mol% of tetramethylethylenediamine was added as an accelerator and the ice bath was removed. After 15 h, a hydrogel was formed, which was then ground into small pieces using a mortar before drying the pieces at 70 °C in a vacuum oven for at least 2 d.

Hydrogels with different degrees of crosslinking between 0.3 and 3.4 mol% and degrees of neutralization between 2.5 and 75 mol% were synthesized (see Table 1). Additionally, an industrial superabsorber named RD 474 from Procter & Gamble (Schwalbach, Germany) was evaluated as larger quantities were available.

The DN is given as the ratio of moles of the base (NaOH) to the total moles of ionizable groups (Equation (7))

(7) $$\mathrm{DN}=\frac{n(\mathrm{base})}{n\left(\mathrm{ionizable monomer}\right)}\times 100\mathrm{mol}\%$$

The DC is determined by the amount of crosslinking agent present in the polymer network relative to the amount of monomer molecules (see Equation (8))

(8) $$\mathrm{DC}=\frac{n(\mathrm{crosslinking agent})}{n(\mathrm{monomer})}\times 100\mathrm{mol}\%$$

The hydrogels were characterized by the degree of swelling *Q* as determined by Equation (9).

(9) $$Q=\frac{m(\mathrm{hydrogel},\mathrm{swollen})}{m(\mathrm{hydrogel},\mathrm{dry})}$$

*Osmotic Engine ‐ Principle of Operation*: The dry superabsorbent polymer was swollen in a 4.3 wt% NaCl solution until the equilibrium degree of swelling was reached. This specific concentration was chosen as a model for seawater since they have the same ionic strength *I*_c_ of 0.729 mol kg^−1^.^26^ The hydrogel was then exposed to deionized water and the expansion under load was measured every minute using an electronic distance gauge (MarCator 1086, Mahr, Göttingen, Germany) or a ruler. The obtained power was normalized to 1 kg dry polymer mass.

**Figure 7:**
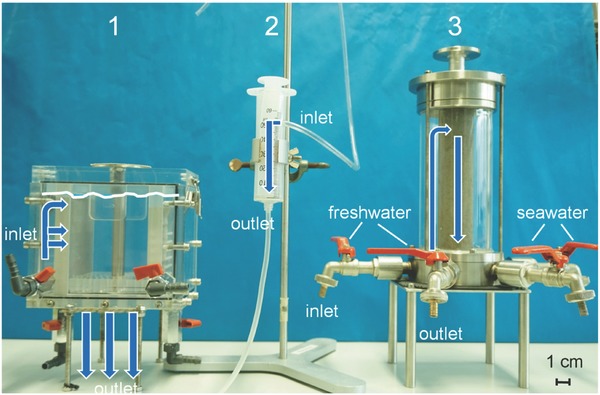
Different Osmotic Engine Prototypes: 1) large scale (≈10 g dry polymer mass), 2) small scale (≈0.25 g dry polymer mass), 3) porous sintered metal tube (≈1 g dry polymer mass). The arrows show the water flow direction through the different prototypes.

*Osmotic Engine Set Ups*: Various osmotic engines were built for testing the power generated by different hydrogels as a function of the engine construction (see **Figure** **7**). Prototype 1 was constructed for testing up to 10 g dry polymer mass and the lifting height of the hydrogel was measured using an electronic distance gauge. It is based on an acrylic glass box with a piston surface area of 81 cm^2^. In Prototype 2, small amounts of ≈0.25 g dry polymer mass can be tested. A 50 mL syringe pump with an area of 7 cm^2^ is the base of this prototype. Prototype 3 is made for tests of 1 g dry polymer mass and contains a porous sintered metal tube inside an acrylic glass tube. Its piston surface area is 12.5 cm^2^. The lifting heights of prototypes 2 and 3 were measured with a ruler because the spring constant of the electronic distance gauge was too high, which led to systematically lower values for the determined energy production.

## Conflict of Interest

The authors declare no conflict of interest.

## Supporting information

Supplementary

Supplementary
