# Supplementary material for: Osmotic Engine: Translating Osmotic Pressure into Macroscopic Mechanical Force via Poly(Acrylic Acid) Based Hydrogels
Source: Adv Sci (Weinh). 2017 May 30;4(9):1700112. doi: 10.1002/advs.201700112 (PMC5604390; doi:10.1002/advs.201700112)
Supplement: Supplementary file 1 — Supplementary [file ADVS-4-na-s001.pdf]

Copyright WILEY-VCH Verlag GmbH & Co. KGaA, 69469 Weinheim, Germany, 2017.

ADVANCED SCIENCE
Open Access

Supporting Information

for *Adv. Sci.,* DOI: 10.1002/advs.201700112

Osmotic Engine: Translating Osmotic Pressure into Macroscopic Mechanical Force via Poly(Acrylic Acid) Based Hydrogels

*Lukas Arens, Felix Weißenfeld, Christopher O. Klein, Karin Schlag, and Manfred Wilhelm**

Supporting Information

Osmotic Engine: Translating Osmotic Pressure into Macroscopic Mechanical Force via Poly(acrylic acid) Based Hydrogels

*Lukas Arens, Felix Weißenfeld, Christopher O. Klein, Karin Schlag, Manfred Wilhelm**

Movie S1: Time lapse recording of several swelling and shrinking cycles. In the experiment, 10 g of a commercial hydrogel (RD 474), consisting of particles in the size of 370 – 670 µm, were alternately exposed to deionized water and a 4.3 wt% NaCl solution to lift a weight of 1.5 kg. In the video, the time lapse is 1:375, resulting in a swelling-shrinking time of about 60 minutes.
